# Toward Enhanced Aerosol Particle Adsorption in Never‐Bursting Bubble via Acoustic Levitation and Controlled Liquid Compensation

**DOI:** 10.1002/advs.202300049

**Published:** 2023-03-26

**Authors:** Xiaoliang Ji, Pingsong Jiang, Yichen Jiang, Hongyue Chen, Weiming Wang, Wenxuan Zhong, Xiaoqiang Zhang, Wei Zhao, Duyang Zang

**Affiliations:** ^1^ School of Physical Science and Technology Northwestern Polytechnical University Xi'an 710129 P. R. China; ^2^ School of Marine Science and Technology Northwestern Polytechnical University Xi'an 710129 P. R. China; ^3^ Xiong'an Institute of Innovation Xiong'an 071899 P. R. China; ^4^ State Key Laboratory of Photon‐Technology in Western China Energy International Scientific and Technological Cooperation Base of Photoelectric Technology and Functional Materials and Application Institute of Photonics and Photon‐technology Northwest University Xi'an 710127 P. R. China

**Keywords:** acoustic levitation, bubble, bubble stability, liquid interfaces, particle adsorption

## Abstract

Bubbles in air are ephemeral because of gravity‐induced drainage and liquid evaporation, which severely limits their applications, especially as intriguing bio/chemical reactors. In this work, a new approach using acoustic levitation combined with controlled liquid compensation to stabilize bubbles is proposed. Due to the suppression of drainage by sound field and prevention of capillary waves by liquid compensation, the bubbles can remain stable and intact permanently. It has been found that the acoustically levitated bubble shows a significantly enhanced particle adsorption ability because of the oscillation of the bubble and the presence of internal acoustic streaming. The results shed light on the development of novel air‐purification techniques without consuming any solid filters.

## Graphical abstract

This work reports a new approach to stabilize bubbles that by using acoustic levitation combined with controlled liquid compensation the bubbles can remain intact permanently. The acoustically levitated bubble shows a significantly enhanced particle adsorption ability which shed light on the developing of novel air‐purification techniques without consuming of any solid filters.

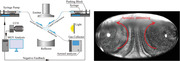

## 1 Introduction

Bubble films play important roles in many natural and industrial processes because they provide unique boundaries for the related heat and mass transfer.^[^ ^1^ ^]^ For instance, bubbles can catalyze chemical reactions to detoxify polluted water, thereby increasing the efficiency of water treatment processes.^[^ ^2^ ^]^ A single bubble is used to measure trace levels of sulfur dioxide, due to the increased conductance of bubbles when exposed to low concentrations of sulfur dioxide gas.^[^ ^3^ ^]^ It can also be used to detect methamphetamine in aerosols by electrical analysis of the bubble wall.^[^ ^4^ ^]^ Because of the excellent adsorption ability of the gas–liquid interface, air bubbles were used for adsorption of aerosol particles,^[^ ^5^ ^]^ which showed the promising application of bubbles for air purification.

Regarding these applications, stable bubbles with retarded bursting are highly desired. However, the lifetime of the conventional “soap” bubbles is usually only a few seconds, because of gravity‐induced liquid drainage,^[^ ^6^, ^7^ ^]^ which is dependent on the viscosity of the solution, the type of surfactants, and bubble size.^[^ ^8^, ^9^, ^10^ ^]^ To enhance the bubble lifetime, various types of approaches have been utilized to restrain the liquid drainage in the bubble film, such as introducing surfactant,^[^ ^11^, ^12^ ^]^ electrolyte,^[^ ^13^ ^]^ and colloidal particles.^[^ ^14^ ^]^ However, these approaches inevitably cause “contamination” of the bubble because of the introduced stabilizer. Alternatively, the bubbles or foams can also be stabilized by sound field using acoustic levitation,^[^ ^15^, ^16^ ^]^ which provides a contact‐free stabilization mechanism to the bubbles or foams, through suppression of the liquid drainage.^[^ ^17^ ^]^

Until now, bubbles of sufficiently long lifetime and without any chemical stabilizers are still a technical challenge. In the present work, we propose technique to fabricate never‐bursting bubbles via acoustic levitation and controlled liquid compensation. Owing to the elimination of capillary waves on the bubble surface by the liquid compensation, the acoustically levitated bubbles can remain intact permanently. The never‐bursting bubble shows enhanced aerosol particle adsorption and provides a promising platform for air purification.

## 2 Results and Discussion

### 2.1 Superstability Arising from Sound Field

**Figure 1:**
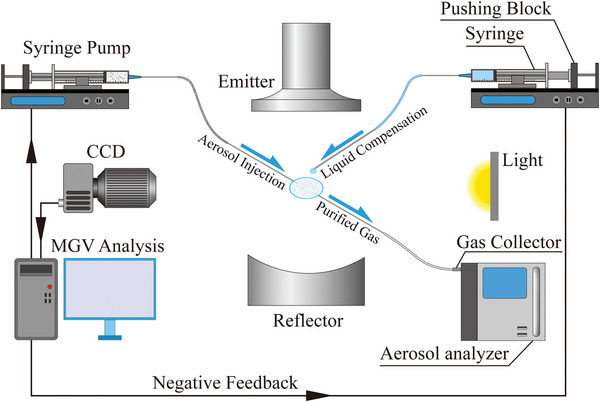
Schematic illustration for the setup of bubble formation, liquid compensation, and air purification. A computer‐controlled system was combined to a single‐axis acoustic levitator, in which the bubble can remain intact permanently. The everlasting bubble can be utilized for aerosol particle adsorption.

**Figure 2:**
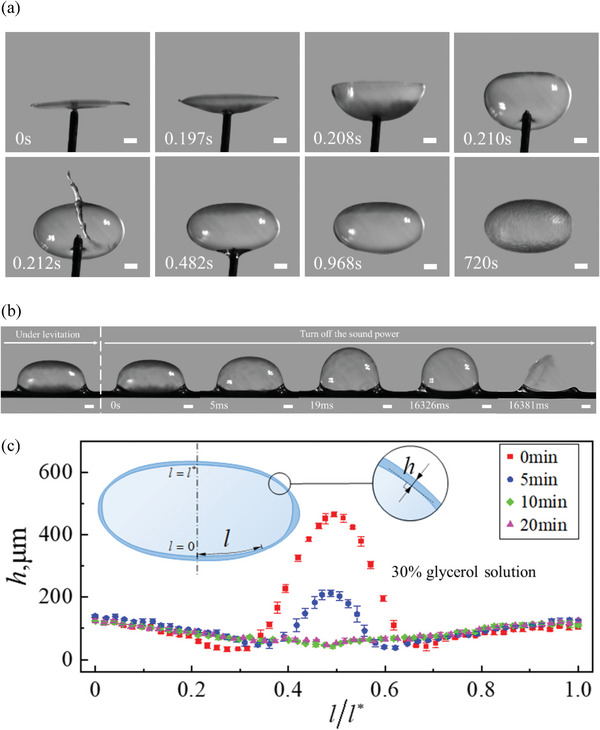
Bubble formation and extraordinary stability under acoustic levitation. a) Bubble formation by dragging a SDS film (10 µL) during levitation. The bubble can remain intact for more than 720 s in the sound field. b) Bubble rupture occurs within 17 s after turning off the levitator, see also Movie S2 (Supporting Information). c) The film thickness distribution of a bubble (30 wt% glycerol solution) under acoustic levitation, showing no notable liquid drainage has occurred, error bars represent standard deviation of the mean for triplicate experimental data. The scale bars correspond to 1 mm.

In this work, the drops and bubbles were levitated and manipulated with a single‐axis acoustic levitator (**Figure** **1**, see also the Experimental Section). The bubble was made via the acoustic resonance approach,^[^ ^18^, ^19^ ^]^ in which the acoustically levitated drop was flattened into a thin film by enhancing sound intensity. The liquid film was dragged by a needle from its center to form a sound cavity (**Figure** **2**a; Movie S1, Supporting Information), which resonated with the sound field, thus leading to cavity inflation and bubble formation. The bubbles can be fabricated in a noncontact manner, and the approach is suitable to different liquids ranging from aqueous solutions to oils.^[^ ^18^ ^]^ Most importantly, the levitated bubble exhibits extraordinary stability and super‐long life. For instance, a glycerol bubble can remain intact for hours. For a sodium dodecyl sulfate (SDS) bubble, it can last over tens of minutes, more than one order of magnitude longer than that without sound stabilization (Figure 2a,b). Moreover, the thickness *h* of the bubble film at its top (*l* = l*) and bottom (*l* = 0) remained constant during levitation (20 min) (Figure 2c), indicating no significant drainage occurred in the levitated bubble, which is in distinct contrast with the traditional bubble.^[^ ^20^ ^]^ The maximum thickness of the bubble film appeared at the equatorial region (*l*/l* = 0.5), where the sound potential well was located and resulted in the liquid accumulation. The thickness reduction at the equator region is probably due to evaporation, where the evaporation rate is fastest.^[^ ^21^ ^]^

**Figure 3:**
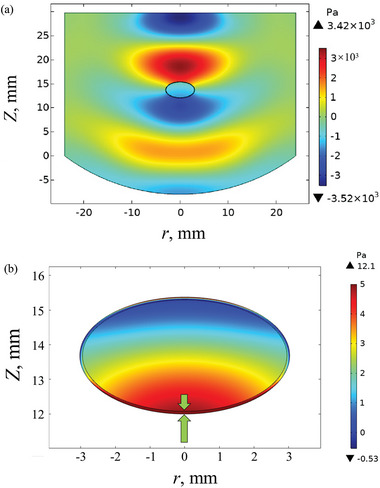
Sound field simulation. a) The sound pressure in the levitator and the levitated bubble. b) Acoustic radiation pressure on the outer and inner surfaces of the levitated bubble.

The ultrasound field not only provides the levitation force to balance bubble gravity but also brings a stabilization mechanism to the levitated bubble which suppress the gravity‐induced drainage. To understand the sound‐caused stabilization, the sound field in the levitator and the acoustic radiation pressure *P*_A_ exerted on the bubble surface were calculated (**Figure** **3**). Owing to the compressibility of the bubble, the acoustic impedance of the bubble surface is greatly reduced,^[^ ^22^, ^23^ ^]^ which enhance the transmittance of sound wave as compared with incompressible air–liquid interface. Therefore, a noticeable sound field was evident inside the levitation bubble (Figure 3a). This led to the presence of non‐negligible *P*_A_ on the inner surface (Figure 3b). *P*_A_ exerts on both the outer and inner surfaces of the levitated bubble, resulting in a squeezing effect (indicated by the arrows in Figure 3b) to the bubble film, which greatly suppressed the liquid drainage. In addition, the acoustically levitated bubble was in the total energy minimum among gravity, sound potential, and surface tension. Liquid drainage would change the thickness distribution of the bubble film, which may lead to energy increase in sound potential and surface energy.^[^ ^17^ ^]^ In other words, liquid drainage is energetically unfavorable.

Surfactants do have a positive effect on the stability of the bubbles because of the retard drainage caused by interfacial rheology brought by surfactants.^[^ ^12^ ^]^ However, in the present study liquid drainage in the bubble film has been largely suppressed by acoustic radiation pressure. In this case, the stabilization effect of surfactant was overshadowed by sound field. In addition, the bubble stability was not influenced by the sound frequency if it can provide sufficient levitation force.^[^ ^16^ ^]^ But under higher frequency, only smaller bubbles can be stably levitated since the appropriate sample size for levitation is determined by the sound wavelength.

The acoustic power plays a crucial role in levitation stability. On one hand, the equilibrium shape of the levitated bubble and local curvature of the bubble film are determined by the competition between surface tension and acoustic radiation pressure:*P_i_* − *P_A_* = *σ*∇ · **n**,^[^ ^24^ ^]^ where *P*_i_ is the internal pressure, **n** is the normal vector on the bubble surface, and ∇ · **n** is the total local curvature. This indicates that the shape and the profile (thickness distribution) of the bubble film would be adjusted upon the variation of acoustic power. Moreover, sufficient high sound intensity often results in atomization,^[^ ^25^ ^]^ which eventually leads to bubble rupture. On the contrary, too low sound intensity generates insufficient acoustic radiation force and the levitation would be easily disturbed. Therefore, in the experiments, the sound intensity was adjusted to an optimized level against both atomization and external disturbance.

### 2.2 Evaporation‐Induced Rupture

**Figure 4:**
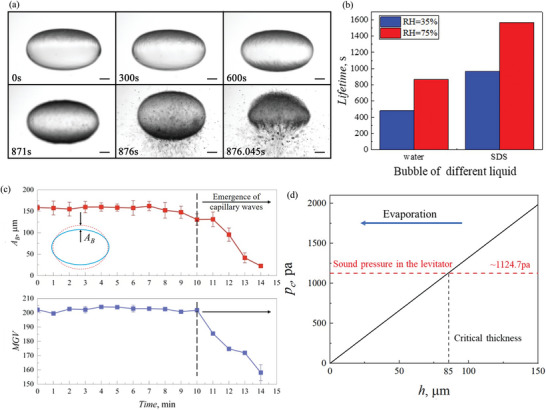
Destabilization of the acoustically levitated bubble. a) Rupture of a SDS bubble. After levitation for 876 s, intense capillary waves emerged due to evaporation, which caused atomization and led to bubble rupture (Scale bars = 1 mm). b) Bubble lifetime enhanced by increasing relative humidity. c) Bubble dynamics from mode‐2 shape oscillation to capillary waves. Upon evaporation, the oscillation amplitude *A*_B_ begins to decrease once capillary waves appeared, which can also be evidenced by the decrease in MGV of the bubble image. Error bars indicate standard deviation. d) The critical sound pressure *p*_c_ for exciting capillary waves decreases with the thinning of the liquid film.

Since the sound media for acoustic levitation is air, evaporation of the levitated liquid is inevitable.^[^ ^21^, ^26^ ^]^ Moreover, the existence of acoustic streaming around the levitated drop may facilitate its evaporation, especially at the drop equatorial region,^[^ ^21^ ^]^ which is also confirmed by the experimental observation in present work (Figure 2c). An acoustically levitated bubble (with short and long radius of 2.1 mm and 3.7 mm) became unstable after evaporation for tens of minutes at room temperature 25 °C and humidity RH = 35%, and tremendous tiny droplets were atomized from the bubble surface, which eventually led to the rupture of the bubble (**Figure** **4**a; Movie S3, Supporting Information). On the contrary, by reducing the evaporation rate under higher relative humidity (from 35% to 75%), the bubble life was significantly enhanced (Figure 4b). These results indicate that liquid evaporation may be the main mechanism responsible for the destabilization and rupture of the acoustically levitated bubble.

Under stable levitation, the bubble gained an equilibrium shape determined by acoustic radiation pressure, surface tension, and the pressure in the liquid film. However, the levitated bubble may exhibit a shape oscillation under external perturbation.^[^ ^18^ ^]^ It has been evidenced that the bubble for different liquids showed a two‐mode oscillation with frequency *f* ≈ 100 Hz in the sound field (Movie S4, Supporting Information), which was independent of the liquid types. Higher mode oscillations have never been observed in the present study, because in the sound field the levitated bubble behaves more like an elastic balloon rather than a droplet.

The natural frequencies *f_n_* of these eigenmodes can be written as^[^ ^27^ ^]^

(1) $$f_{n}=\frac{1}{2\pi }{\left\{\frac{\sigma \left(n^{2}-1\right)\left(n+2\right)}{R^{3}\left(2n+1\right)\rho_{0}}\right\}}^{\frac{1}{2}}$$

where *n* and *R* are the oscillation mode and equivalent radius of the bubble, *σ* is the surface tension of the liquid, and *ρ*_0_ is the density of air. The frequency for two‐mode oscillation of the bubbles in the present work was calculated in the range from 216 to 323 Hz, depending on the surface tension of the liquids. This indicates the levitated bubble was not in a free oscillation but a quadrupole shape oscillation resonated with the acoustic radiation pressure.^[^ ^28^, ^29^ ^]^ The oscillation amplitude *A*_B_ was ≈150 µm, and tended to decrease after 10 min evaporation (Figure 4c), suggesting the emergence of capillary waves.

The capillary waves on the acoustically levitated bubbles are Faraday waves excited by parametric resonance with sound field.^[^ ^30^ ^]^ The critical sound pressure *p*_c_ to excite the capillary waves can be written as^[25]^

(2) $$p_{c}∼2^{\frac{4}{3}}\rho \nu {\left(\frac{\rho }{\sigma }\right)}^{\frac{1}{3}}\omega^{\frac{5}{3}}h$$

where *ω* = 2*πf*_c_ is the angular frequency of the capillary waves. The frequency *f*_c_ of these waves was 12.6 kHz, about half the sound frequency, and with a wavelength *λ*(∼(*σ*/*ρ*)^1/3^ *f*_c_^−2/3^) around 109 µm.^[^ ^30^ ^]^ The density *ρ* and kinematic viscosity *υ* of SDS solution in the experiment are 994 kg m^−3^ and 1.157 × 10^−6^ m^2^ s^−1^, respectively, and the surface tension *σ* is 32.55 mN m^−1^. Equation (2) can be simplified as

(3) $$p_{c}∼1.32\times 10^{7}h$$

This indicates *p*_c_ decreases linearly with the thinning of the film (Figure 4d). In the present study, the sound intensity of the levitator is about 155 dB with a corresponding sound pressure ≈1124.68 Pa. This suggests once the film thickness was thinned upon evaporation below the critical thickness of 85 µm, the sound pressure became greater than *P*_c_ and capillary waves were excited (Figure 4a). The capillary waves were further enhanced upon film thinning and led to atomization eventually.

Once capillary waves appeared, owing to its scattering effect, the transmittance for white light showed an obvious decrease and the photos became darker (Figure 4a, 871 s). Based on the image analysis, the mean gray value (MGV) of the photos exhibited a notable decrease (Figure 4c). Similar experimental observations were also found in water bubbles (Figure S2, Supporting Information). The results indicate that capillary waves causing a decrease in MGV is a general observation, which may be used to detect the destabilization of the levitated bubble.

### 2.3 Dynamic Liquid Compensation and Stability Recovering

**Figure 5:**
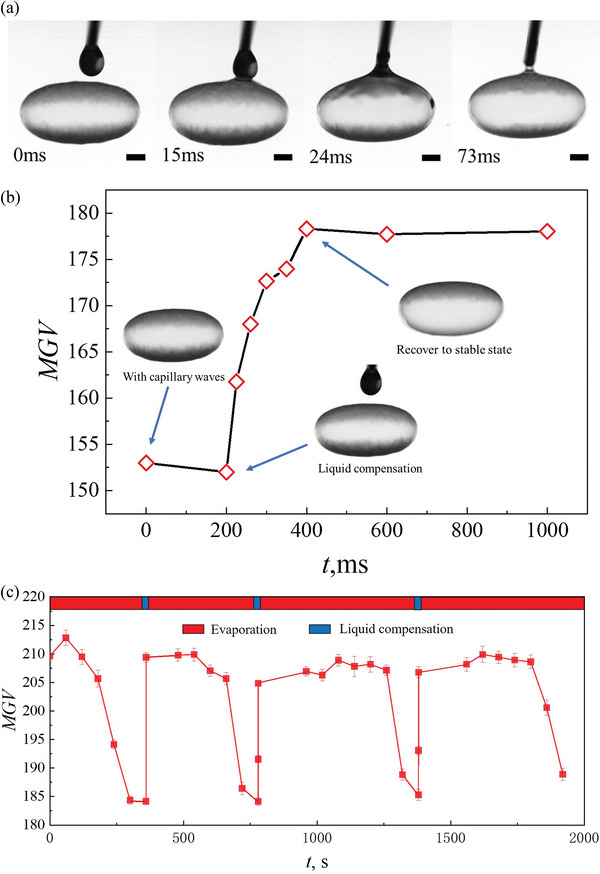
Liquid compensation toward everlasting bubble. a) Droplet‐bubble coalescence during liquid compensation (Scale bars = 1 mm). b) Bubble stability recovery caused by liquid compensation. c) Evaporation‐liquid compensation cycles accomplished by a computer‐controlled system, which enable long time stability of the levitated bubble. Error bars represent the standard deviation.

Film thinning caused by evaporation is the main destabilization mechanism for the acoustically levitated bubble. Owing to the existence of acoustic streaming, suppression of evaporation is hard to be accomplished. To prolong the lifetime of the levitated bubble, we proposed a liquid compensation approach, in which droplet of liquid was introduced to the sound field and coalesced with the levitated a bubble. Driven by surface tension and acoustic force,^[^ ^31^ ^]^ the coalesce was finished within tens of milliseconds (**Figure** **5**a). After liquid compensation, the thickness of the bubble film was increased. As a result, the excited capillary waves were damped (Figure 5b). Meanwhile, the levitated bubble recovered to stable levitation state as evidenced by the increased MGV. This approach can damp capillary waves, and in turn prevent atomization and bubble rupture.

Since the status of the levitated bubble, i.e., the emergence of capillary waves on bubble surface can be detected by the MGV value (Figure 4c), we further built a computer‐controlled liquid compensation system with the negative feedback from MGV analysis. The control law is

(4) $$D=\left\{\begin{array}{c} k\left(G-G_{t}\right)\;\;\;\;\;\;\;\;\;\;\;\;\;\;\mathrm{if}\left(G>G_{t}\right)\\ 0\;\;\;\;\;\;\;\;\;\;\;\;\;\;\mathrm{if}\left(G\leq G_{t}\right) \end{array}\right.$$

where *D* is the duty cycle of pulse width modulation (PWM) control for the pump motor, *G* is the current MGV value, and *G_t_* is the threshold value (185 in the present work) before the emergence of capillary waves. By utilizing the liquid compensation system, a SDS bubble can remain intact for approximately half an hour, which has experienced three evaporation–compensation cycles. The results hint the acoustically levitated bubble can remain intact permanently as long as the working time of the system.

### 2.4 Air Purification Using Acoustically Levitated Bubble

**Figure 6:**
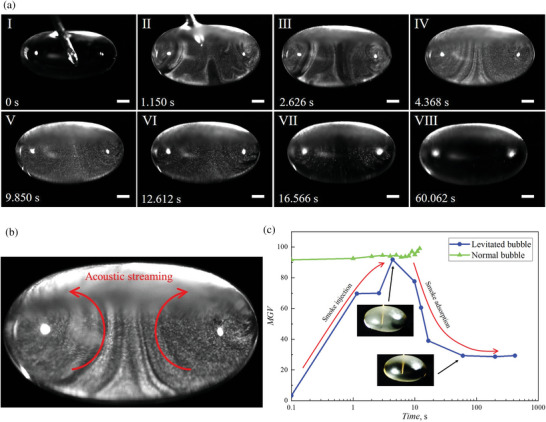
Enhanced aerosol particle adsorption by acoustically levitated bubble. a) Snapshots showing the injection smoke and adsorption of the smoke particle in a levitated SDS bubble (Scale bars = 1 mm). b) Acoustic streaming inside the levitated bubble. c) Comparison between temporal MGV for an acoustically levitated bubble and a traditional half‐bubble.

Since the air–liquid interfaces have excellent trapping ability for micro/nanoparticles,^[^ ^32^ ^]^ we thus explored to use the liquid film of the acoustically levitated bubble as a collector for aerosol particles. Because of the superstability arising from sound field,^[^ ^17^ ^]^ the levitated bubble was able to survive from puncturing by a needle yet remain intact (**Figure** **6**a). Aerosol gas was then injected to the bubble within 2 s. There existed a torus‐like acoustic streaming in the bubble, which was clearly demonstrated by the aerosol particles as tracers (Figure 6b). Within 20 s, the flow patterns became invisible indicating that most of the particles have been absorbed by the bubble film. This hints the injected aerosol gas has been effectively purified.

The aerosol particle adsorption can be monitored by the transparency of bubble, which was analyzed using the MGV of the bubble photos (Figure 6c). To elucidate the effect of acoustic levitation on particle adsorption, we compared the temporal MGV of an acoustically levitated bubble and a traditional bubble without sound field. Under acoustic levitation, significant adsorption can be accomplished in tens of seconds. Whereas without the pressure of sound field, no noticeable adsorption occurred in the entire life of the bubble, ≈12 s. There results highlight that acoustic levitation not only enables the long lasting life of the bubble, but also greatly enhanced the adsorption of injected aerosol particles.

It remains to explain why an acoustically levitated bubble has extraordinary adsorption ability. For the particle adsorption, it depends on two processes: 1) Stage I, the particles reach and contact the bubble surface driven by the acoustic streaming. 2) Stage II, once in contact with the bubble surface, the particles are trapped at the air–liquid interface. For Stage II, the liquid type as well as SDS concentration plays a role because of the varied surface tension, viscosity, etc. However, it should be noted that for the aerosol particles all the liquids can enable sufficient fast trapping and irreversible adsorption.^[^ ^5^ ^]^ Therefore, the key issue should be addressed is to accelerate the contact between particles and bubble surface in Stage I.

We have found that the acoustic streaming inside the bubble, which was aroused from bubble oscillation,^[^ ^33^ ^]^ plays an essential role to accelerate the contact between particles and bubble surface, which is not sensitive to the bubble size and liquid type. The injected particles circulated with the acoustic streaming, during which the particles impinged onto the bubble at a speed ≈0.01 m s^−1^. Once the imping particles contact with the air–liquid interface, they can be adsorbed within 0.1 ms.^[^ ^34^ ^]^ Moreover, the levitated bubble usually rotates around the vertical axis,^[^ ^18^ ^]^ which further enhance the mass transfer between the bubble surface and the injected aerosol gas.

Based on the facts that acoustically levitated bubble has extraordinary particles adsorption ability and the never‐bursting bubble technique, we proposed a potential air‐purification system by using a levitated bubble as a filter element (Figure 1). Since the bubble can remain intact permanently via liquid compensation, thorough particle adsorption can be realized with sufficient adsorption time. Our work sheds light on the development of novel air purification techniques, without consuming any solid filters.

## 3 Conclusion

In summary, we have achieved extraordinary stability of bubbles by using acoustic levitation combined with controlled liquid compensation, through which the bubbles can remain unburst permanently. In addition to sound field induced bubble stability, the critical novelty of our technique lies in the suppression of capillary waves on the bubble surface via liquid compensation thus preventing the atomization and bubble rupture. The extraordinary stability and ever‐lasting life of the bubble enable them to be used for aerosol particle adsorption. It has been found that the adsorption in the acoustically levitated bubble is significantly enhanced due to the bubble oscillation and the internal acoustic streaming. Based on these results, we have proposed an air purification system, in which polluted air was injected into the levitated bubble, purified by the bubble films and the clean air was collected in a controlled manner. Our work provides a reference for the fabrication of never‐bursting bubbles and sheds light on the developing of novel air cleaners using bubble film as filters.

## 4 Experimental Section

### Bubble Formation

A single‐axis acoustic levitator (SonoRh‐1, Shengli Ltd., Nangjing, China) comprising a transmitter and reflector (Figure 1) was used to levitate the droplets and bubbles, and the working frequency of the levitator was 20.5 kHz. A light source and high‐speed camera (Photron Fastcam Mini UX100, Japan) were combined to record the dynamic process. The bubble was obtained through droplet‐to‐bubble transition via the acoustic resonance mechanism. In the experiment, a droplet was injected into the ultrasonic field using a microsyringe connected to a computer‐controlled pushing block, and the levitated droplet was deformed into a disc‐shaped liquid film by reducing the emitter–reflector distance. The bubble was formed by dragging the film from its center using a copper wire.^[^ ^18^ ^]^ The bubble was trapped at one of the sound pressure nodes in the levitator. The sound field was adjusted to a suitable intensity to enable satisfactory levitation stability in which the levitation position did not show noticeable horizontal or vertical shift.

### Measurement of the Bubble Film Profile

To measure the thickness of the bubble film, the levitated bubble was fluorescent stained with Rhodamine B and illuminated by a laser sheet generated by a continuous‐wave Nd:YAG laser with 532 nm wavelength. The laser sheet cut longitudinally through the central axis of the bubble. In order to make the measurement of bubble film thickness more accurate, the laser sheet was focused by a cylindrical lens with long focal length. The thickness of the laser sheet at the focus was 500 µm, which is sufficiently smaller than that of the field depth of the imaging system (2.9 mm). Thus, the position and distribution of the illuminated film were determined by the laser sheet, not the camera. On the other hand, the depth of focus of the cylindrical lens is 28 mm, which is larger than the diameter of the bubble, to ensure the thin laser sheet has uniform thickness at the focus. The image of bubble film profile was captured by a high‐speed camera (Photron Fastcam Mini UX100, Japan). The thickness of bubble film was measured using a software called ImageJ, after calibration in the software.

### Image Analysis

To build the relation between the bubble image and the associated physical/chemical processes, it is necessary to obtain the MGV of the images. The images were captured by the high‐speed CCD camera. To ensure the reproducibility of MGV, the photographic process was conducted at a fixed illumination condition by using a stable LED light source. A real‐time processing algorithm running on the control computer was written with OpenCV library to get the bubble contour. The photos were transferred into a 256 gray‐scale image via gray processing, where the gray value is 0 for black pixels and 255 for white pixels. The MGV of the bubble was obtained from the integration of the corresponding pixels in the contour area.

### Liquid Compensation

Liquid compensation to the acoustically levitated bubble was realized by depositing a droplet using a microsyringe connected with a computer‐controlled peristaltic pump (Figure 1). The compensation process was controlled based on the negative feedback according to the change in MGV of the levitated bubble. The MGV was obtained via image analysis and input to the computer in real time. Once the MGV of the bubble decreased to the threshold MGV *G*_T_, a push of the peristaltic pump was triggered and a droplet of ≈5 µL was squeezed out. It is worth noting that in order to avoid possible destabilization caused by the liquid compensation, the introduced droplet was moved slowly to contact the bubble to realize a moderate coalescence. The threshold MGV *G*_T_ was set below the MGV corresponding to the appearance of capillary waves.

### Air Purification

The aerosol gas (the size of the particles ranging from 0.1 to 1 µm, and the average effective density of the particles is 1120 ± 40 kg m^−3[^ ^5^ ^]^) was generated by an aerosol generator (LB‐3300, LuBo Ltd, Qingdao, China) and injected into the acoustically levitated bubble using a microsyringe connected with a computer‐controlled peristaltic pump. The needle was inserted from the top of the bubble, and penetrated the bubble film without rupture because of the superstability arising from sound field. Aerosol particle adsorption was detected by the changes in bubble transparency, which is represented by the MGV of the images. The purified air was collected and analyzed by an aerosol analyzer (PC‐3A, XinYe Ltd., Qingdao, China).

### Sound Field Simulation

The sound field in the ultrasonic levitator and the sound radiation pressure on the bubble surface were calculated using the commercial finite element software COMSOL Multiphysics 5.6. A 2D axisymmetric model (frequency domain) was used in the calculation, and the physical field control grid was set as the “finer grid” built in the software. The simulation range was determined by the geometry of the levitation space. The boundary conditions of the levitation zone were set separately, i.e., the normal acceleration at the emitter was calculated as 4*π* ^2^ *f_a_* ^2^ *A* (where the frequency *f_a_* of the acoustic levitation was 20.5 kHz and the amplitude *A* at the emitter was 14.5 µm as measured by a dial gauge), the reflector was set as a rigid boundary, and the side wall of the levitation zone was set as a radiation boundary.

In the levitator, the acoustic medium was air (density *ρ*_0_ = 1.18 kg m^−3^, sound velocity *c*_0_ = 346.12 m s^−1^). According to the levitation position and shape of bubbles in the ultrasonic field, the corresponding bubble model was constructed, and its boundary condition was set as a continuous boundary, which is determined by the acoustic impedance mismatch between the two phases separated by the interface. However, for the calculation of the sound field inside the bubble, the impedance of the bubble film (air–water interface) cannot be set according to the impedance mismatch between water and air because of the oscillation or even resonance of bubbles in the sound field.^[^ ^22^, ^23^ ^]^ This suggests that the acoustic impedance of the bubble film, which can be estimated from the mean density of the bubble over its whole volume instead of the density of liquid in the film, may be appreciably reduced.

The sound pressure and sound pressure level were obtained by solving the Helmholtz equation in the acoustics module of the software. According to King's theory,^[^ ^35^ ^]^ the acoustic radiation pressure *P_A_* can be calculated as

(5) $$\langle P_{A}\rangle =\frac{1}{2\rho_{0}c_{0}}\langle p_{1}^{2}\rangle -\frac{\rho_{0}}{2}\langle u_{1}^{2}\rangle$$

where *p*_1_ is the sound pressure and *u*_1_ is the particle (parcel of fluid) velocity of the medium.

The weight of the levitated sample is balanced by the acoustic radiation force *F*_A_, which is the integral of *P*_A_ exerted on both the inner and outer surfaces of the bubble

(6) $$F_{A}=\;\int \int \;P_{A}ds=mg$$

where *m* is the mass of the bubble, and *g* is acceleration of gravity.

## Conflict of Interest

The authors declare no conflict of interest.

## Supporting information

Supporting Information

Supplemental Movie 1

Supplemental Movie 2

Supplemental Movie 3

Supplemental Movie 4

## Data Availability

The data that support the findings of this study are available from the corresponding author upon reasonable request.
