# Supplementary material for: Toward Enhanced Aerosol Particle Adsorption in Never‐Bursting Bubble via Acoustic Levitation and Controlled Liquid Compensation
Source: Adv Sci (Weinh). 2023 Mar 26;10(19):2300049. doi: 10.1002/advs.202300049 (PMC10323653; doi:10.1002/advs.202300049)
Supplement: Supplementary file 1 — Supporting Information [file ADVS-10-2300049-s001.pdf]

ADVANCED SCIENCE
Open Access

Supporting Information

for *Adv. Sci.*, DOI 10.1002/advs.202300049

Toward Enhanced Aerosol Particle Adsorption in Never-Bursting Bubble via Acoustic Levitation and Controlled Liquid Compensation

*Xiaoliang Ji, Pingsong Jiang, Yichen Jiang, Hongyue Chen, Weiming Wang, Wenxuan Zhong, Xiaoqiang Zhang, Wei Zhao and Duyang Zang**

Supplemental materials

Toward enhanced aerosol particle adsorption in never-bursting bubble via acoustic levitation and controlled liquid compensation

Xiaoliang Ji[1], Pingsong Jiang[1], Yichen Jiang[2], Hongyue Chen[1], Weiming Wang[3], Wenxuan Zhong[1], Xiaoqiang Zhang[1], Wei Zhao[4], Duyang Zang[1]*.

[1] *School of Physical Science and Technology, Northwestern Polytechnical University, Xi'an 710129, China*

[2] *School of Marine Science and Technology, Northwestern Polytechnical University, Xi'an 710129, China*

[3]*Xiong'an Institute of Innovation, Xiong'an 071899, China*

[4]*State Key Laboratory of Photon-Technology in Western China Energy, International Scientific and Technological Cooperation Base of Photoelectric Technology and Functional Materials and Application, Institute of Photonics and Photon-technology, Northwest University, Xi'an 710127, China*

*Correspondence: dyzang@nwpu.edu.cn

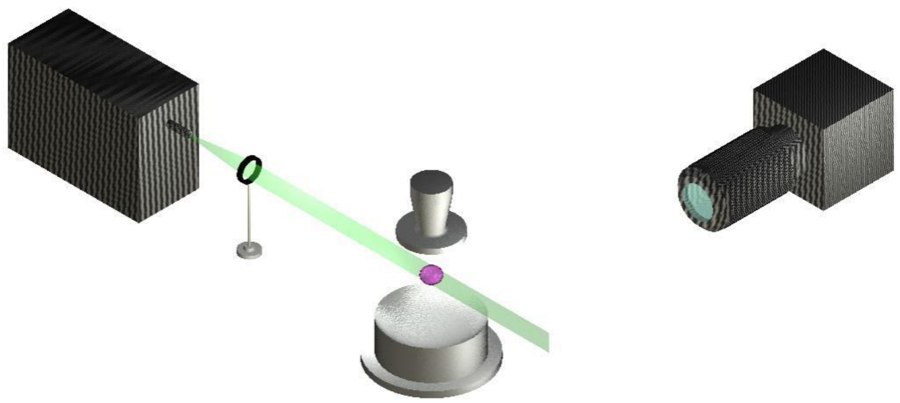

Fig.S1 Schematic illustration of the instrument used to measure the bubble film profile.

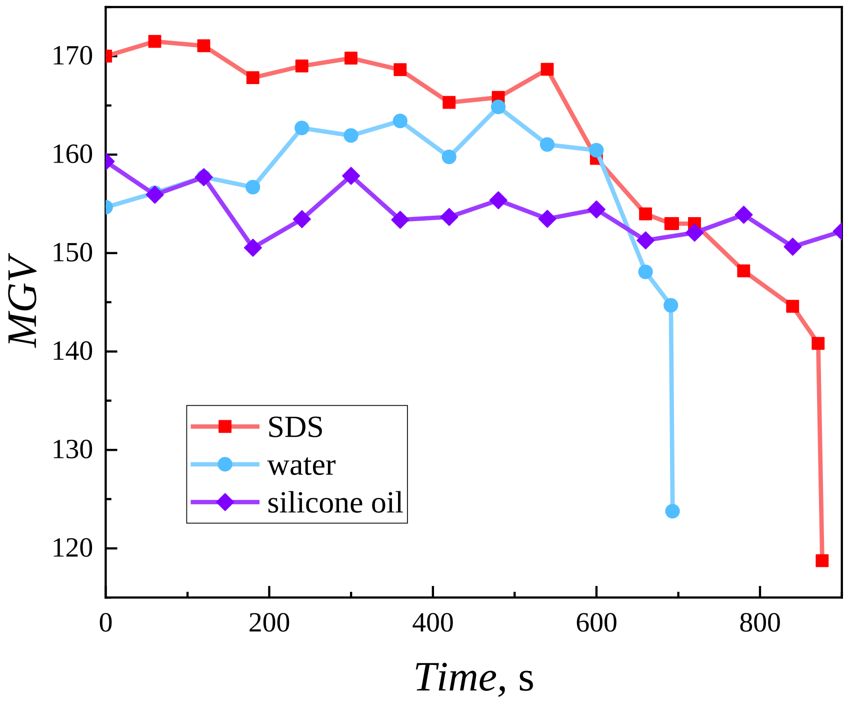

Fig.S2 The relationship between the capillary wave intensity of different bubble surfaces and the evaporation time.

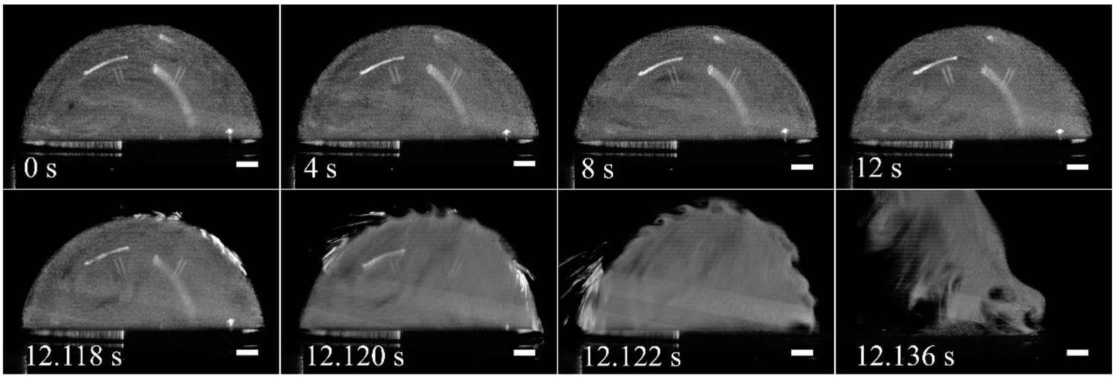

Fig.S3 The adsorption of smoke particles in normal bubbles is inefficient, and due to the short lifetime of bubbles, particles are released into the air again with the burst of bubbles.
